# Tumor Hypoxia: How Conventional Histology Is Reshaped in Breast Carcinoma

**DOI:** 10.3390/ijms26094423

**Published:** 2025-05-06

**Authors:** Péter Juhász, Gábor Méhes

**Affiliations:** Department of Pathology, Faculty of Medicine, University of Debrecen, Nagyerdei krt. 98, H-4032 Debrecen, Hungary; peter.juhasz@med.unideb.hu

**Keywords:** hypoxic stress, adaptation, breast carcinoma, molecular subtypes, prognosis

## Abstract

Intratumoral hypoxia is common in any form of malignancy initializing focal necrosis or tumor cell adaptation. Hypoxia inducible factor-1-driven reprogramming favors the loss of tumor cell proliferation (quiescence) and partial cellular reversion, induces stemness and/or mesenchymal-like features in the exposed tumor areas. The characteristic hypoxia-driven tumor cell phenotype is principally directed to reduce energy consumption and to enhance survival, but the gained features also contribute to growth advantage and induce the reorganization of the microenvironment and protective mechanisms against external stress. The hypoxia-induced phenotypic changes are at least in part reflected by conventional morphology in breast carcinoma. Intratumoral variability of classical morphological signs, such as the growth pattern, the histological grade, cell proliferation, necrosis, microcalcification, angiogenesis, and the immune cell infiltration is also related with the co-existence of hypoxic areas. Thus, a deeper understanding of hypoxia-activated mechanisms is required. The current paper aims to summarize the major tissue factors involved in the response to hypoxia and their potential contribution to the breast carcinoma phenotype.

## 1. Introduction

Adequate tissue perfusion is a major requisite of cancer progression. Principally all active cellular processes depend on the delivery of abundant amounts of oxygen and nutrients. However, the actual oxygen supply is influenced by both delivery and utilization and thus highly variable in time and space. The intratumoral partial oxygen pressure (pO_2_) is determined by local factors, such as capillary density, interstitial hydrostatic pressure, tumor mass, increased tumor metabolic activity, and O_2_ consumption.

The intolerable degree of hypoxia ends up in massive tumoral necrosis, frequently observed in high-grade cancers, such as invasive breast carcinoma (BC).

Early sub-lethal forms of hypoxic stress, on the other hand, induce a wide spectrum of adaptive changes to reduce energy consumption and to enable the use of any kinds of alternative ATP sources to support survival. The net result of hypoxia (death or survival) is strongly dependent on the intensity (a drop in the pO_2_ level) and on the duration (acute, intermittent, chronic) of hypoxia. Highly proliferative tumors are typically composed of areas with variable blood perfusion, resulting in well and poorly oxygenated areas with an alternating pattern.

A long-lasting, low O_2_ supply generally results in the suppression of anabolic activities, thus inducing cellular silencing. However, adaptive changes fall under the oxygen-sensitive proactive control of hypoxia inducible factors 1 and 2 (HIF-1 and HIF-2) [1,2]. HIF-1 is one of the most studied transcription regulators in hypoxia, which acts through the upregulation of the alpha (HIF-1α) subunit of the protein dimer. The active HIF-1α/HIF-1β dimer is enabled to bind and initialize genetic reprogramming through hypoxia responsive elements (HRE) distributed throughout the genome [3]. Accumulation of HIF-1α was found to be the major driver of adaptation in cancer cells coordinating the mechanisms of the metabolic switch and neutralization of intracellular acidosis and further inducing tumor cell stemness and resistance to apoptosis [4,5]. The moderate efficacy of the glucose utilization program (the Warburg-effect) is further jinked by the upregulation of glucose transporters (e.g., GLUT1), metabolic enzymes (e.g., lactate dehydrogenase and hexokinase II), and by the activation of defense mechanisms controlling intracellular acidosis, such as Na+/H+ exchanger-1 (NHE-1), carbonic anhydrase IX (CAIX) and XII (CAXII), and monocarboxylate transporter 4 (MCT 4). In the meantime, CAIX evolved as a major hypoxia marker the clinical effect of which on metabolic acidosis is acknowledged [6,7].

Transient hypoxia, on the other hand, may directly activate cellular features through parallel regulatory pathways, e.g., overexpression of cell cycle regulators (e.g., cyclins D1, A, B1 and E2F) or induction of growth factor receptors, such as epithelial growth factor receptor (EGFR) and vascular endothelial growth factor receptor (VEGFR) [8]. A further long-term effect of tissue hypoxia is the induction of new vessel formation (angiogenesis) [1]. To restore effective tissue perfusion and create an optimal microenvironment the production and release of proangiogenic growth factors, like the vascular endothelial growth factor (VEGF), placental growth factor (PlGF), angiopoietins, but also basic fibroblast growth factor (bFGF), platelet-derived growth factor-β (PDGFβ) is stimulated [9,10]. Taken together, the adaptative features generally contribute to the survival and the progression in hypoxia-trained cancer cells. Consequently, common morphological features like the growth pattern, the histological grade, the cell proliferation, necrosis, microcalcification, angiogenesis, and the immune cell infiltration may be fundamentally affected. Interestingly, activation of hypoxia-related mechanisms shows differences in histological and molecular BC subtypes.

Next, we try to summarize significant hypoxic changes influencing tissue morphology in BC and evaluate the role of adaptive mechanisms responsible for histological heterogeneity in diagnostic samples.

## 2. Biochemical and Genomic Effects of Hypoxia on Breast Carcinoma

Tumoral necrosis can be considered an obvious result of local hypoxia. However, the expression and distribution of HIF-1-related survival factors appear to have a fundamental effect on tissue patterns and tumor growth characteristics. The expression of hypoxia-related markers HIF1, GLUT1, and CAIX were studied in relation to necrosis [11]. According to these early results, focal hypoxia by marker expression can be stated in 1/3 of breast carcinomas lacking necrotic morphology.

The establishment of molecular subtypes luminal A and B, HER2+ and triple-negative breast carcinoma (TNBC) molecular subtypes and their phenotypic correlates resulted in a differential picture also reflecting the biological heterogeneity in hypoxia response. In vitro measurement of surrogate metabolic activities under hypoxia presented that the oxygen consumption rate (OCR) raised in luminal A and B type cells and decreased in the HER2+ and the TNBC group. In reverse, the extracellular acidification rate (ECAR) was lower in the luminal A/B group and higher in the HER2+/TNBC groups, indicating a differential adaptive behavior, as shown by the gene expression findings described below [12].

Cellular adaptation following the variations of oxygen tension resulted in molecular signatures of non-specific genetic markers in BC-derived cancer cells, like in other cancer types [13]. Gene expression studies demonstrated that the luminal phenotype (ER+/PgR±) was prominently associated with the activation of glucose uptake (*GLUT1*, *SLC2A1*, *HSPA5*, *HYOU1*), gluconeogenesis (*FBP1* and *2*, *IDH2*, *G6PD*), glycolysis, glucose–lactate conversion, and the pentose phosphate pathway, while the mitochondrial respiration remained relatively preserved. These features were even more extended in the luminal B molecular subtype (ER+/PgR±, HER2+). In contrast, the Her2+ subtype BC showed differential MAPkinase and mTOR signaling, activation of the toll-like receptor pathway, further to the increased glucose consumption, LDH-A expression, and lactate production [14]. According to a similar study, genes involved in glycolysis and lactate metabolism were more activated in luminal-like cells while cytoskeletal control associated with cell migration was affected in the basal A-like cells [15].

TNBC representing the most unfavorable disease category is not just genomically heterogeneous but is more than any other category associated with improper oxygenation and hypoxia driven metabolic switch [16,17]. The TNBC subtype was characterized by gene expression involving epithelial–mesenchymal transition (*VIM*, *TGFb1 COL3/6/8*, *PLOD2*, *CD44*) and enabling cell migration, e.g., actin cytoskeletal organization (*ACTB*) or matrix-metalloproteinases (*MMP2/14*) under the control of HIF-1 [14].

A differential proteomic profile of luminal and basal-type BC was reported, mostly relying on hypoxia and metabolic stress-related protein expression. The expression analysis of a panel of 33 stromal and secreted proteins evaluated here indicated strong prognostic and predictive differences further to the established molecular subtypes [18].

## 3. Hypoxia-Related Biomarkers in Breast Carcinoma

A coordinated overexpression of selected hypoxia genes was observed in high-grade and human epidermal growth factor receptor-2-positive (HER2+) tumors which could also be related to cancer recurrence [19,20]. Major HIF-1 responsive genes included VEGF, GLUT1, and CAIX [21]. The role of HIF-1α was extensively studied and its overexpression was found to be an independent adverse prognostic feature in BC [22]. Other studies concluded that hypoxia-inducible acidity regulator MCT4, in interaction with CAIX, indicates unfavorable outcome in triple-negative BC [23,24]. The negative prognostic effect of erythropoietin-receptor (EPO-R) overexpression in BC in response to HIF-1 could also be stated [25]. However, while HIF1, GLUT1, VEGF, EPO, and MCT4 were reported as promising biomarkers in the past, their clinical utility is still not widely accepted in BC diagnostics.

The family of carbonic anhydrases gained a more special interest [26]. CAIX was warranted as an evolving prognostic biomarker in breast carcinoma [27]. A major cause for the surpassing role of CAIX was its simple demonstration by tissue immunohistochemistry [28]. Successive clinical studies independently reported a statistically significant negative prognostic effect of CAIX expression on BC survival [29,30,31]. CAIX was also proposed to be a biomarker of tumor oxygenation in vivo in breast cancer by innovative in vivo imaging approaches [32,33].

**Figure 1:**
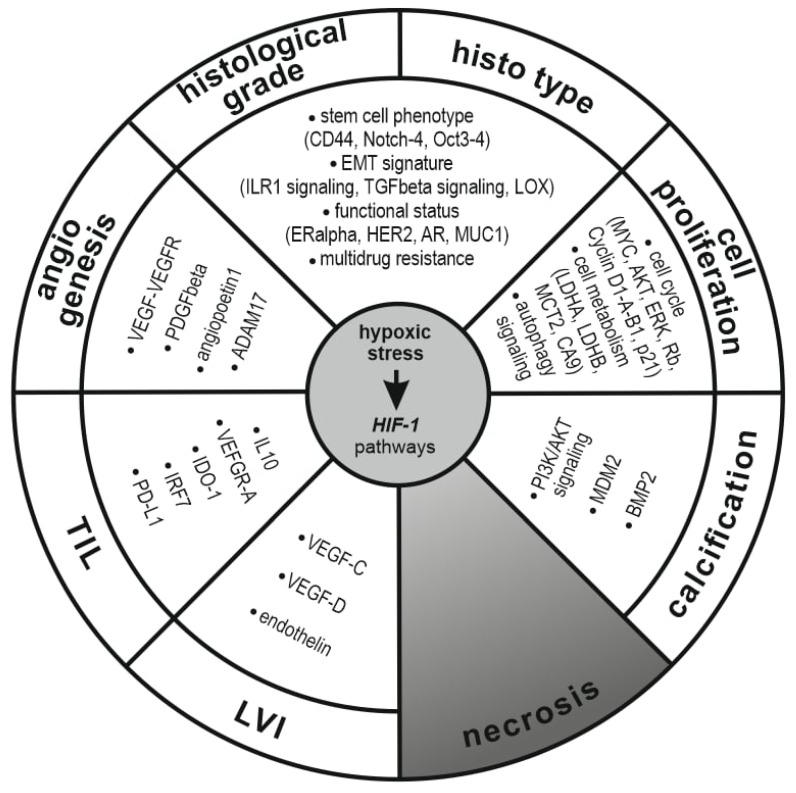
Major effects of tumor hypoxia on molecular pathways and their potential association with classical tumor morphology defined by conventional histological parameters in breast carcinoma.

The hypoxic breast tissue microenvironment has also been consistently identified as a feature that promotes tumor survival, the resistance to therapy and supports the metastatic process in BC [16,34,35]. The specific effect of intermittent hypoxia more than chronic hypoxia was reported in association with pro-metastatic phenotype and cytokine secretion [36]. The stromal expression of HIF-1, MCT4, and CAIX indicative of increased glycose metabolism in hypoxic areas proved to be associated with unfavorable disease outcomes in invasive ductal carcinoma [37,38]. As a special stromal feature, tumor-directed immune responses are also profoundly affected, causing the potential failure of natural and therapy-induced tumor cell killing [39]. The major effects of tissue hypoxia on tumoral compartments are summarized in Figure 1.

## 4. Classic Histological Features and Prognostic Scoring of Breast Carcinoma

The clinical prognosis of breast cancer is primarily defined by clinicopathological criteria defined after surgery. These include the tumor size, histological grade and the success of the primary treatment (surgical resection margins, regressive features following neoadjuvant treatment). In addition, the response to biological therapy can be predicted by tumor features, such as the estrogen receptor (ER), progesterone receptor (PR), and HER2 expression, also provided by histology. Based on the most recent histological, biological, and molecular updates, the WHO (World Health Organization) Classification continuously reviews BC subtypes and prognostic categories [40]. The standardized reporting covers all clinically established histological criteria to support clinical appreciation and to enable therapeutic decision making. Report formats are issued by international clinical cancer organizations (College of American Pathologists/CAP, International Collaboration on Cancer Reporting/ICCR) [41,42,43].

It is of basic impact that most histological features to be reported show topographical variability and are under the direct influence of tumor perfusion and hypoxic stress. In fact, the morphological complexity and general appearance of breast carcinoma is strongly related to hypoxia. Features like cell proliferation, differentiation, growth pattern, necrosis, scar formation and fibrosis, morphological regression, and high-grade transformation all include aspects of hypoxia driven cellular reprogramming (Figure 1). In the following section, we make an attempt to summarize the effects of hypoxia on histological features covered by standardized breast carcinoma reporting.

## 5. The Effect of Hypoxia on Conventional Histological Features of Breast Carcinoma

### 5.1. Histologic/Molecular Subtype

HIF-1-regulated pathways seem to have a general effect on breast cancer progression and survival [14,20,44,45]. However, according to the diverse morphological and molecular features, BC subtypes are also expected to differentially respond to tissue hypoxia, determined by genomic, epigenomic, transcriptomic, and immune/microenvironmental programs. The differential response to hypoxia in the molecular BC subtypes was already discussed above. HIF-1 induced stemness, the activation of a mesenchymal-like phenotype, changes in the tumor–stroma ratio, and increased cellular plasticity go in parallel with the loss of mammary-type ductal/glandular differentiation and a more aggressive histological type, according to earlier studies [46,47].

Some of the phenotypic changes following hypoxia-induced reprogramming have specific clinical relevance. As such, the repression/loss of endocrine steroid receptor (e.g., ER alpha) signaling in receptor-positive tumors was reported [48,49]. Moreover, the gain of anticancer drug resistance due to the activation of HIF-1 hypoxia signaling was also stated in hormone receptor positive BC, also explaining the low response rate to androgen receptor (AR) inhibitors [50,51].

### 5.2. Histological Grading

Microscopic grading in BC is based on the Nottingham/modified Bloom and Richardson Score evaluating tumor differentiation related parameters defined by microscopy [52]. Grading of the invasive part of the tumor includes the evaluation of tubule/gland formation, the nuclear pleomorphism (including size and hyperchromatic staining), and the mitotic rate [53]. The scoring of each of these elements is based on standardized criteria, however, standardization is challenged by tissue heterogeneity and interobserver variability.

The cancer cell morphology is principally influenced by metabolic changes caused by reduced oxygen and nutrient delivery resulting in “degenerative” alterations of both the cytoplasm and the cell nucleus of the stressed tumor areas. Cytoplasmic changes (abundant eosinophilic, as well as clear, foamy or granular appearance) and tubular disorganization is to be expected in association with intermittent hypoxia. Tumor cell nuclei frequently show regressive changes (shrinkage, loss of nucleoli, heterochromatin clumping) to be observed in parallel with higher grade pleomorphism, referring to a hypoxic/metabolic environment. HIF-1α expression was found to be associated with higher histologic grade in parallel with other adverse prognostic factors, like high Ki67 expression, negative ER status and positive lymph node status [22]. CAIX overexpression was also reported to be associated with higher tumor grade and epitope shedding [54]. Thus, a histologic grade and especially its intratumor variability are supposed to be related to partial/non-lethal forms of hypoxia. To increase accuracy, the grading of the “advancing tumor areas” was recommended, as the most viable and preserved part of the sample is less likely to suffer from hypoperfusion. However, the selection is difficult to control, especially in needle-core biopsy specimens.

### 5.3. Mitotic Counts and Cell Proliferation Activity

The mitotic rate is the most objective feature of the grading system as mitotic counts can be determined in standardized microscopic field areas. More recently, the values should be given per mm^2^ (introduced in the WHO 2019 Breast Tumor classification). The counting of the fields with the highest densities is recommended (hot spot, leading edge).

One of the most accepted markers representing tumor activity is the cell proliferation marker Ki67. This classic cell-cycle chaperone protein is expressed in large quantities in cycling cells, thus providing a reliable basis for the estimation of the proliferative fraction by immunohistochemistry, including BC [55,56]. Probably, the most widely used antibody clone is distributed under the name Mib-1. Extended studies clearly justified its prognostic role and predictive potential with enhanced efficacy especially in early-stage ER-positive BCs [57,58]. The preanalytical and the interobserver (post-analytical) limitations of the Ki67 index determination was extensively discussed and thresholds with clinical impact were analyzed in detail [59,60,61].

The evaluation of mitotic counts and the Ki67-rate reflects the general activity while low-grade/regressive compartments suffering from hypoperfusion may escape interest. With our current knowledge, however, low-pleomorphic/low-proliferative areas represent a clinically underrated reserve potential. Chronic or recurrent hypoxia induces long term tumor-cell dormancy and stem-cell like features. Energetically exhausted tumor foci most likely represent a transient condition but at the same time relish protection triggered by HIF-1α and by the local environment. As a result, a pool of resting cells, also called the “hypoxic niche”, is generated [62]. This specific condition not simply supports prolonged survival and drug resistance but also allows the selection of temporarily suppressed cell clusters with tumor-repopulating features.

### 5.4. Necrosis–Fibrotic Foci

Tumor necrosis is an established morphologic hallmark of aggressive tumor phenotype, typically seen in high-grade or basal-type carcinomas of the breast [63,64,65]. Intraductal necrosis is a characteristic feature of ductal carcinoma in situ, however, the impact of necrosis at these sites is confused by the localized, finite O_2_ distribution of the early neoplastic lesion [46].

Both the amount and the distribution of the necrotic areas was found to be significant in BC, although with a lot of controversies. A negative effect of necrosis on aggressiveness is generally accepted, but results clarifying the relation of these simultaneous, virtually contradicting features are still missing. Necrosis frequently occurs in solid tumor areas with high proliferative activity because of local hypoxia due to delayed angiogenesis, vessel compression or disruption, increased intratumoral pressure, and vascular tumor invasion. Demarcation of the necrotic zone with a surprisingly sharp transition to preserved tumor parenchyma is frequently seen. Alternating necrotic and viable tumor areas suggest a perfusion dependent distribution, where areas at larger distance from the vascular stroma more prominently suffer from lethal hypoxic damage. As expected, the peri-necrotic zones are highlighted by hypoxia markers and signatures (e.g., CAIX overexpression) [29,30]. A significant portion (approx. 30%) of non-necrotic BC also presents with hypoxia-related changes, accounting for the general attendance of transient, reversible hypoxic stress in the life and fate of cancer cells [11].

Fibrotic foci (FF) are well-defined scar-like lesions located centrally which are supposed to be the late consequence of intratumor necrosis in high-grade metastatic BC. In contrast, diffuse tumoral fibrosis is more likely a result of complex oncogenic dysregulation of the tumor stroma. The degree of FF is a characteristic feature of BC, occurring mostly in primary invasive ductal carcinoma. Extended fibrosis was related to an adverse outcome. FF were reported to be associated with HIF-1α, CAIX, and Glut-1 overexpression indicative of chronic hypoxia signaling in the central part of the tumor [66,67].

### 5.5. Microcalcification

Microcalcification (MC) could be identified in up to 50% of invasive BC samples [68,69]. As widely accepted, MC occurs because of hypoxia induced focal tumor necrosis. As such, MC can be found within both the DCIS and the invasive component, but even non-neoplastic parts of the breast lesion can be involved and become prominent following treatment. MC is made of crystalline calcium oxalate or hydroxyapatite deposited around necrotic cellular debris, according to the conventional views [70]. More recently the role of mesenchymal-type phenotypic switch and a consecutive osteogenic activity was suggested. The gain of ectopic bone-forming capacity during breast carcinogenesis was repeatedly discussed [71,72]. Behind the unclear biochemical mechanism of calcium deposition, a direct association with hypoxic stress and HIF-1α pathways in invasive tumors could be established [69]. Evidence is growing that HIF-1α-mediated transactivation induces osteogenic differentiation and focal calcification in a hypoxic tissue microenvironment [73,74].

### 5.6. Lymphovascular Invasion (LVI)

LVI is a typical feature of BC with aggressive phenotype and is usually associated with increased proliferative activity, high histologic grade. and loss of hormone receptor expression. The clinical significance of LVI is also expressed by the proposed independent prognostic role negatively affecting patient survival [75,76]. As a logical consequence, lymph node positivity and higher risk of recurrence is to be expected in LVI-positive cases [77]. Tumor necrosis is a frequent simultaneous finding in cases with LVI providing a connection with hypoxia. Although not systemically studied, some of the hypoxia-related biomarkers, e.g., erythropoietin receptor (EpoR) and vascular endothelial growth factor-C and -D (VEGF-C, VEGF-D) were reported to be upregulated in association with LVI [78,79]. A direct correlation of these factors with HIF-1α could be stated [80,81].

### 5.7. Angiogenesis

The angiogenic phenotype of the tumor is defined by both cancer and stromal cells sensitive to hypoxia. The intensity of blood vessel growth is regulated through the expression of paracrine factors, such as vascular endothelial growth factor (VEGF), basic fibroblast growth factor (bFGF), angiopoetin 1, angiopoetin 2, and erythropoetin [82]. Induced by hypoxic stress, mammary stromal fibroblasts and inflammatory cells (mainly macrophages) physiologically produce angiogenic factors and downregulate angiogenesis inhibiting mechanisms to increase vascular density and improve tissue perfusion. Hypoxia-related angiogenesis is generally considered as a reaction to intermittent/chronic hypoperfusion.

In addition to the environmental response, cancer cells also frequently gain the capacity to overexpress VEGF unrelated to hypoxia that was also found to enhance intratumoral vessel density [83,84]. Tumor-related neovascularization, on the other hand, may also induce cell proliferation in a paracrine fashion, e.g., through insulin-like growth factor-1 (IGF-1) and platelet-derived growth factor (PDGF) produced by endothelial cells.

Tumor microvessel density (MVD) was suggested as an objective measure of angiogenesis [85,86]. However, the dynamic change in oxygenization and angiogenetic stimuli result in significant spatial (and temporal) differences and thus in high-grade heterogeneity of parenchymal blood vessel formation. Due to its uneven distribution, the determination of MVD is subject of continuous investigation [87,88]. Endothelial cells of newly formed tumor vessels were highlighted more specifically by the expression of nestin, an intermediate filament protein of progenitor cell nature [89]. Further to the vessel quantity, the specific aberrant shape of newly generated capillaries indicates prognostic information [90]. In summary, the actual angiogenetic phenotype reflects a highly variable biological spectrum in BC. Intermittent/chronic hypoxia and acquired subclonal genetic/epigenetic evolution of the tumor most probably act synergistically, resulting in high-level spatial complexity.

### 5.8. Tumor-Infiltrating Lymphocytes (TIL) and Immune Function Modulation Under Hypoxia

The number and distribution of immune effector cells infiltrating the tumor parenchyma was shown to have prognostic and predictive impact also in BC [91,92,93]. Intra- and peritumoral lymphocyte counts could be easily accessed in conventional H&E-stained sections, while a more accurate immune cell composition can be determined by immunohistochemistry.

The percentage of intratumoral lymphocytes compared to the total number of tumor cells can be easily stated by microscopy, the evaluation is made in increments of 10% for simplicity. However, the exact determination and standardized evaluation remains challenging and has been repeatedly debated [94,95]. The distribution of tumoral lymphocytes is unfortunately uneven throughout the tumor; therefore, the estimation of the average lymphocyte counts is favored over calculations in hot-spot areas. Despite these analytical issues the comparison of BCs with low and high TIL values allowed to differentiate immunologically anergic and active tumors in a simple fashion. TIL was found to be associated with a favorable therapeutic response after neoadjuvant therapy in breast carcinoma [96,97]. A more specific determination of the tumor-infiltrating CD8+ T-cells tied over some of the interpretative issues and a predictive role on disease survival could be stated [98].

The inflammatory reaction induced during cancer progression is dynamically regulated and influenced in any direction by complex genetic and environmental features [99]. Both the immune cell reception and cancer cell immunogenicity were proposed to be strongly affected by the hypoxic/metabolically compromised environment [100]. As for many other functions, the HIF-1-directed reprogramming results in high variability depending on the time, intensity and site of hypoxia. As such, low oxygen tension was shown to negatively regulate MHC class I expression of cancer cells in a HIF-1 dependent manner [101]. Significant changes in tumor immune microenvironment were also reported, including the upregulation of immune checkpoints—such as PD-L1 (CD276) and NRP1—and activation of B-cells and CD8+ T-cells in invasive tumors following hypoxia [102].

The negative effect of tissue hypoxia on the anti-tumor immune functions could also be demonstrated in the clinical context. Gene expression signatures demonstrated significantly better overall survival in hypoxia low/immune high compared to hypoxia high/immune low groups in diverse cancer subtypes. In TNBC, reduced immune responses including low cytotoxic T-lymphocyte infiltration were observed in the latter group, also followed by differentially upregulated CAIX and PD-L1 [103]. In another study, *CA9*, *PGK1*, and *SDC1* gene expression-based hypoxic risk score could efficiently distinguish prognostic categories of BC patients. The expression levels of five relevant immune checkpoint components (*PD1*, *CTLA4*, *TIGIT*, *LAG3*, and *TIM3*) in the hypoxic high-risk group were significantly elevated compared with the low-risk group [104].

The quantity and the distribution of intratumoral immune cells are both matters of change according to the local perfusion. The heterogenous composition of the immune environment could also be related to a regional hypoxia-related gene expression pattern [105]. One of the key issues provided by anaerobic tumor cell metabolism is the increased lactate production resulting in both intra- and extracellular acidosis. The reduced extracellular pH induces a profound reorganization of the tumor micro-environment, also influencing the migration and functional capacities of effector immune cells. In a smaller study our group also found that CD8+ T-lymphocytes and tumor areas with CAIX-expression are mutually exclusive in invasive BC samples. We concluded that obvious CAIX expression significantly reduces CD8+ T-lymphocyte abundance with a negative influence on natural and therapeutic immune response [106]. This observation fits other findings demonstrating a negative impact of hypoxia related immune variables resulting local immunosuppression in BC and other malignancies [106,107,108,109,110]. According to the current concept the failure of immunotherapies may correlate with an “immunologically cold” cancer phenotype, a clinically relevant biological category also triggered by intermittent or chronic hypoxia [111].

The function of tumor-associated macrophages (TAM) in hypoxic environments is less well studied and understood in BC. M2 macrophages contribute to the remodeling of the ECM and support tumor cell invasion. High TAM was generally reported to indicate adverse prognosis. It seems to be established that TAM activity is strongly affected by tissue hypoxia, mainly through the induction of galectin-3 secretion, NF-kB activation, and ROS generation [112]. Further, the release of the vascular growth factor VEGF established a link between TAM, enhanced angiogenesis, and tumoral T-cell infiltration [113,114].

## 6. Conclusions

In BC, distinct molecular programs have been identified which run in parallel: estrogen-responsive, proliferative, hypoxia-induced, inflammation-related. Regressive morphology becomes obvious at the level of the cell nucleus and cytoplasm, of the growth characteristics, and of the proliferative and mitotic activity. A shift in the receptor expression pattern and intercellular interactions, including stromal activation, angiogenesis, and immune cell invasion, are measurable. Adaptive changes primarily directed to reduced energy consumption and enhanced survival result in a characteristic hypoxia-driven tumor cell phenotype with differential biological behavior and protection against external harm.

Hypoxia through activation of the HIF-1 pathway induces glycolysis, angiogenesis, invasion and metastasis, BC stem cell enrichment, and immune escape, all acting as positive selection mechanisms contributing to BC survival.
